# Toward routine health phenotyping: High-throughput prediction of metabolic, immune, and inflammatory biomarkers from milk mid-infrared spectroscopy in early-lactation dairy cows

**DOI:** 10.3168/jdsc.2026-1122

**Published:** 2026-09-18

**Authors:** P.N. Ho, J.E. Hemsworth, C.M. Reich, C.R. Bath, Z. Liu, S. Rochfort, M. Khansefid, M.S. Tahir, M. Haile-Mariam, M.E. Goddard, L.C. Marett, S.R.O. Williams, C.K.M. Ho, M.J. Berkhout, R. Xiang, A.J. Chamberlain

**Affiliations:** 1 Agriculture Victoria, AgriBio, Centre for AgriBioscience, Bundoora, Victoria 3083, Australia; 2 School of Applied Systems Biology, La Trobe University, Bundoora, Victoria 3083, Australia; 3 School of Agriculture, Food and Ecosystem Sciences, Faculty of Science, the University of Melbourne, Victoria 3010 Australia; 4 Agriculture Victoria, Ellinbank, Victoria 3821, Australia

## Abstract

This study evaluated the potential of milk mid-infrared (MIR) spectroscopy, combined with routinely available on-farm variables, for predicting serum metabolic, immune, and inflammatory biomarkers in early-lactation cows. Data included 5,936 blood samples from 4,442 cows across 23 Australian dairy herds, with paired milk MIR spectra and serum measurements for up to 14 biomarkers. Prediction models were developed using partial least squares regression and evaluated using nested 10-fold random cross-validation and leave-one-herd-out validation. The results show that although basic herd-test data, including milk fat, protein, and lactose concentration, as well as on-farm variables, including DIM, calving age, breed, and herd, could predict serum biomarkers, combining MIR spectra with these on-farm variables produced the best overall performance. In random cross-validation, BUN was predicted most accurately (R^2^ = 0.79), and BHB and nonesterified fatty acids (NEFA) showed moderate accuracy (R^2^ = 0.57 and 0.45, respectively). Blood urea nitrogen also showed the strongest external validation performance, with leave-one-herd-out R^2^ = 0.58 and comparable accuracy for predicting records collected after 70 DIM (R^2^ = 0.65). The BHB and NEFA had moderate leave-one-herd-out accuracy but did not transfer beyond early lactation. Most other biomarkers showed low or inconsistent external validation performance. Overall, MIR spectroscopy combined with on-farm variables shows promise for routine prediction of BUN, BHB, and NEFA, which can be used for monitoring and genetic evaluation of, for example, ketosis and energy deficit. Initial random cross-validation results for glucose, bilirubin, and cholesterol were promising, but more data are needed to improve the prediction accuracy and robustness of the predictions.

## Graphical Abstract

**Figure:**
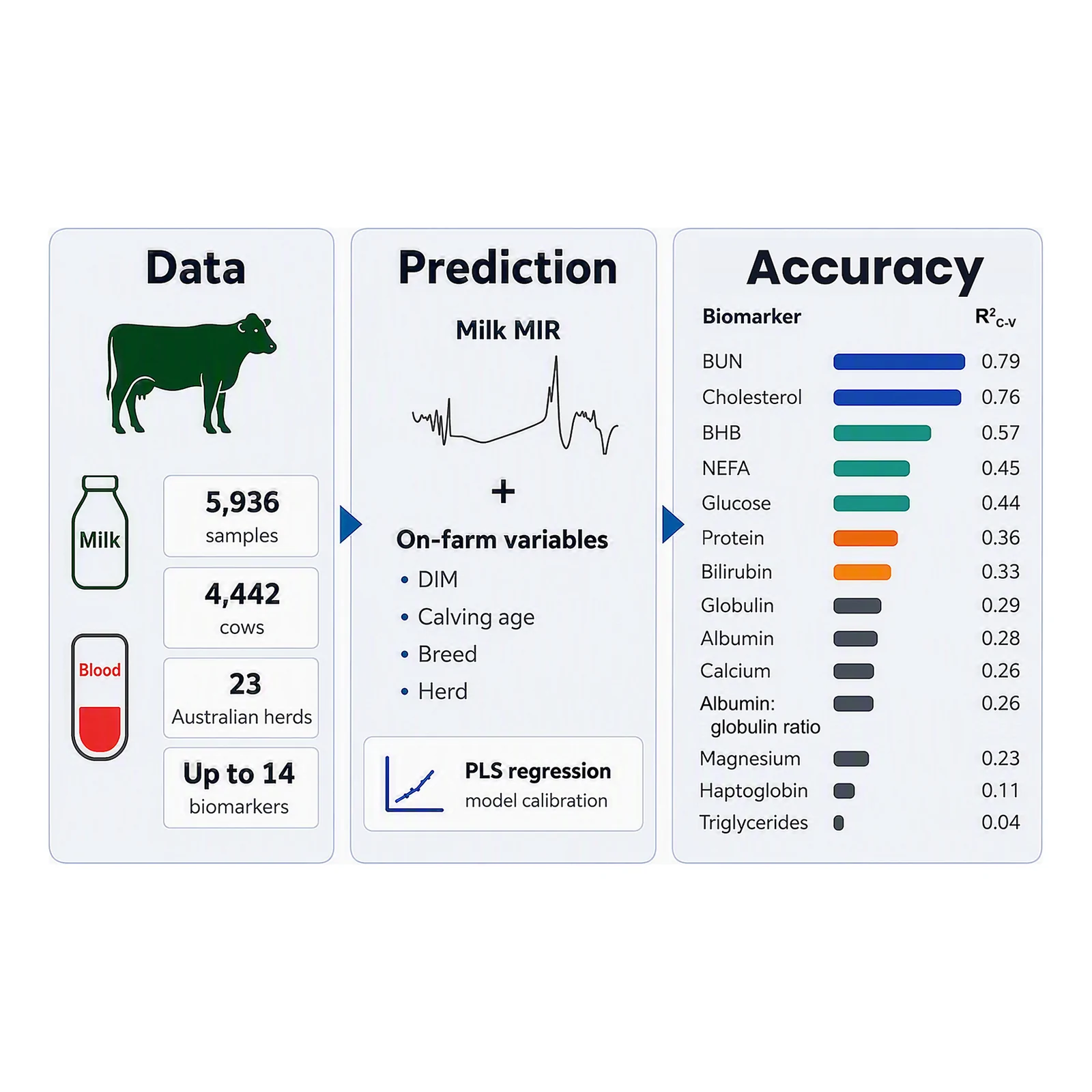

Early postpartum is the most metabolically demanding stage of the dairy cow's productive life and is characterized by rapid shifts in energy balance, liver function, mineral homeostasis, and immune status following the onset of lactation (Overton et al., 2017; Trevisi et al., 2025). These changes increase the risk of metabolic disorders that impair production, health, and reproduction (Kang et al., 2025). The economic impact of impaired transition health is substantial, including increased treatment costs, greater culling risk, and reduced milk production and fertility. Therefore, monitoring metabolic status during early lactation is critical for supporting timely management interventions. In addition, routinely generated metabolic health indicators could provide valuable phenotypes for genomic selection to improve transition cow health (Pryce et al., 2016).

Serum metabolic profiling is widely used to assess the metabolic health and nutritional status of dairy cows. Common serum biomarkers include BHB and nonesterified fatty acids (**NEFA**) as indicators of energy balance and ketosis risk; albumin and BUN as indicators of protein metabolism, inflammatory status, and nutritional adequacy; calcium and magnesium as indicators of macromineral status; and bilirubin, cholesterol, triglycerides, and glucose as measures of hepatic function and lipid and carbohydrate metabolism (Overton et al., 2017). Haptoglobin is also an important acute-phase protein because elevated concentrations reflect systemic inflammation (Lassallette et al., 2026). Routine blood sampling in dairy cows is, however, labor-intensive, costly, and invasive, limiting its feasibility for repeated monitoring at the herd scale.

Milk mid-infrared (**MIR**) spectroscopy has emerged as a promising approach to predict metabolic and health-related biomarkers in dairy cows. Among these, BHB, NEFA, and BUN are the most extensively studied, with reported prediction accuracies (R^2^) from random cross-validation ranging from 0.50 to 0.70, 0.39 to 0.53, and 0.54 to 0.87, respectively (Benedet et al., 2019; Grelet et al., 2019; Ho et al., 2021). Giannuzzi et al. (2023) demonstrated that MIR spectroscopy can predict a broad panel of 29 blood biomarkers (R^2^ = 0.20–0.83), including those related to energy metabolism, liver function, oxidative stress, inflammation, and mineral status. Milk MIR spectroscopy offers a practical alternative to direct blood assays because it is rapid, cost-effective, and already implemented routinely by commercial milk-testing organizations for milk component testing. As such, MIR-based prediction models for health-relevant biomarkers could enable population-level phenotyping without additional sampling costs, facilitating both herd management and large-scale genetic evaluation (Luke et al., 2019a; Dale et al., 2021).

Building on our previous Australian work demonstrating the promise of MIR spectroscopy for predicting BHB, NEFA, and BUN (Luke et al., 2019b; Ho et al., 2021), the objective of this study was to extend the analysis to a broader panel of metabolic, immune, and inflammatory biomarkers in early-lactation cows, including Ca, Mg, total protein, albumin, globulin, bilirubin, cholesterol, haptoglobin, triglycerides, and glucose.

The data used in this study consisted of 5,936 blood samples taken from 4,442 lactating cows of 23 dairy herds located in Victoria, New South Wales, and Tasmania (Australia) between July 2017 and June 2024. This dataset also included previous records from Luke et al. (2019b) and Ho et al. (2021). Cows were in their first to ninth parity with 54.5% being Holstein-Friesian, 4.7% purebred Jersey, and 40.8% crossbred. Their DIM varied from 1 to 305, with 89.5% of samples taken within 70 d after calving.

The blood samples were analyzed for concentrations of BHB, NEFA, BUN, Ca, Mg, total protein, albumin, globulin, bilirubin, cholesterol, haptoglobin, triglycerides, and glucose. Because the data were compiled from multiple research projects, not all samples were analyzed for the same set of biomarkers. The following assays were used: enzymatic kinetic assays for BHB and BUN; enzymatic end-point assay for NEFA; colorimetric complexometric assays using Arsenazo III (pH 6.5) for calcium and Xylidyl Blue for magnesium; the biuret method for total protein; the bromocresol green (pH 4.2) method for albumin; the diazo colorimetric Jendrassik–Grof method for bilirubin; end-point enzymatic colorimetric assays for cholesterol, triglycerides and glucose; and hemoglobin-binding peroxidase activity colorimetric kinetic assay for haptoglobin (Liu et al., 2025). Globulin concentration was calculated as total protein minus albumin and the albumin-to-globulin ratio (**AG**) was also calculated. Serum analyses for samples collected between 2017 and 2020 were conducted at Regional Laboratory Services (Benalla, VIC, Australia) using a Konelab 20XTi automated clinical chemistry analyzer (Thermo Fisher Scientific, Vantaa, Finland). All samples collected thereafter were analyzed at the AgriBio Laboratory, Agriculture Victoria (Bundoora, VIC, Australia), using a ChemWell 2910 Automated EIA and Chemistry Analyzer (Awareness Technology Inc., Palm City, FL), using Catachem Inc. (Oxford, CT) reagents, controls, and calibrations as per the manufacturer's instructions. The AgriBio instrument was calibrated and validated to be consistent with that of Regional Laboratory Services.

Milk samples were collected within 12 h of blood sampling and were analyzed for concentration of fat, protein, and lactose, as well as SCC, using a NexGen Series FTS Combi instrument (Bentley Instruments, Chaska, MN). All milk samples collected in 2017, together with the ones from 2 Tasmanian herds in 2018 were processed at TasHerd Pty Ltd. (Hadspen, TAS, Australia), whereas the remaining milk samples were processed at HICO Pty Ltd. (Maffra, VIC, Australia). In addition to milk composition, MIR spectra generated by the instruments were retained and used in this study.

All animal procedures in this study were conducted in accordance with the Australian Code of Practice for the Care and Use of Animals for Scientific Purposes (NHMRC, 2013). Ethical approvals were granted by the Department of Energy, Environment and Climate Action (DEECA) Agricultural Research and Extension Animal Ethics Committee, who were licensed to conduct animal research in Victoria, New South Wales and Tasmania for all samples collected after April 2020 and the Tasmanian Department of Primary Industries, Parks, Water and Environment (Animal Biosecurity and Welfare Branch, New Town, Tasmania, Australia) for the 2 Tasmanian herds collected in 2018.

Before model development, several mathematical treatments were applied to the serum biomarkers and spectra, using the complete dataset. A logarithmic (10) and a square root transformation were applied to the original records of BHB and NEFA, respectively. This was done because our preliminary analyses (results not shown) indicated that their natural distributions were not normal, which might impair the accuracy of predicting high values in partial least squares regression (Grelet et al., 2016). For the spectra, noisy regions (1,615–1,652 cm^−1^ and 649–925 cm^−1^) characterized by a low signal-to-noise ratio, which is the consequence of a high water absorption and noninformative region (2,998–3,998 cm^−1^), were first removed. Second, to discard the spectra that are potentially outliers, a standardized Mahalanobis distance between each spectrum and the population average was calculated. Then, the spectra with a global distance greater than 3 (n = 26) were considered outliers and eliminated. Last, a first-order Savitzky–Golay derivative was applied to the reduced spectra. The final spectra used for model development consisted of 536 wavenumbers. We also attempted to standardize spectra generated by the TasHerd instrument to those generated by the HICO instrument using 100 identical milk samples and the Piecewise Direct Standardization method (Wang et al., 1991), but this produced results comparable to those obtained using the original spectra. This agrees with Broutin (2016), who showed that the Bentley infrared cell and spectra standardization gave consistent BHB predictions over time and globally. Therefore, original spectra were used for all analyses. The number of records and summary statistics for each biomarker are summarized in Table 1.

**Table 1:** Summary statistics of serum biomarkers (mean ± SD)

Biomarker^1^	DIM ≤70				71 ≤ DIM ≤ 200			
	No. herds	No. cows	No. records	Mean ± SD	No. herds	No. cows	No. records	Mean ± SD
BHB, mmol/L	23	4,387	5,279	0.67 ± 0.46	9	271	625	0.64 ± 0.24
NEFA, mmol/L	23	4,390	5,281	0.51 ± 0.28	9	271	625	0.38 ± 0.29
BUN, mmol/L	23	4,393	5,284	5.34 ± 2.08	9	271	625	5.01 ± 1.94
Calcium, mmol/L	12	2,010	2,756	2.31 ± 0.22	5	258	612	2.38 ± 0.21
Magnesium, mmol/L	12	1,865	2,323	0.97 ± 0.18	5	190	218	0.91 ± 0.29
Protein, g/L	23	4,047	4,848	72.69 ± 6.53	9	271	625	80.02 ± 6.19
Albumin, g/L	23	4,047	4,848	34.34 ± 3.66	9	271	625	34.06 ± 3.46
Globulin, g/L	23	4,047	4,848	38.35 ± 6.52	9	271	625	45.96 ± 7.08
AG	23	4,047	4,848	0.93 ± 0.22	9	271	625	0.77 ± 0.17
Haptoglobin, g/L	17	1,939	1,951	0.20 ± 0.19	6	13	14	0.19 ± 0.14
Cholesterol, mmol/L	3	436	506	2.58 ± 1.14	1	182	209	4.93 ± 0.99
Glucose, mmol/L	8	1,630	2,149	3.32 ± 0.67	3	258	611	3.32 ± 0.35
Bilirubin, μmol/L	3	419	490	3.30 ± 2.53	1	182	209	2.83 ± 3.81
Triglycerides, mmol/L	3	437	508	0.22 ± 0.17	1	182	209	0.27 ± 0.21

1 NEFA = nonesterified fatty acids; AG = albumin-to-globulin ratio.

Because this study focused on monitoring cow health during early lactation and given that relatively few records were available from later lactation (71 ≤ DIM ≤ 200), only records collected within 70 DIM postpartum, representing approximately 90% of the data, were used for model development. Models were developed using partial least squares regression (**PLSR**) implemented with the caret R package (Kuhn, 2008). Model performance was assessed by nested 10-fold cross-validation and leave-one-herd-out validation. In addition, models developed using records collected within 70 DIM were evaluated using records collected after 70 DIM to assess their ability to extrapolate beyond the calibration set.

For nested 10-fold cross-validation, model evaluation and parameter tuning were separated using outer and inner 10-fold cross-validation loops. To avoid information leakage arising from repeated measurements, all records from the same cow were assigned to the same fold in both the outer evaluation loop and the inner tuning loop. In the outer loop, cows were partitioned into 10 folds, with each fold used once as the test set and the remaining 9 folds used for training. Within each outer training set, an inner 10-fold cross-validation was used to select the optimal number of latent variables based on root mean square error (**RMSE**). The model was then refitted to the full outer training set using the selected number of latent variables and evaluated on the held-out outer test fold. Predictors were centered to a mean of 0 and scaled to a variance of 1 before model fitting. This procedure was repeated 50 times, and final performance estimates were calculated as the mean of the 50 replicate means across the 10 outer folds.

For leave-one-herd-out validation, one herd at a time was excluded from model training and used as an independent validation set. Models were trained using data from the remaining herds and evaluated on the excluded herd. This process was repeated until every herd had been validated once. Herd was fitted as a categorical predictor using one-hot encoding. Because the excluded herd was not present in the training data, no corresponding herd indicator was available for validation records from that herd. Consequently, all herd-indicator variables for the excluded herd were set to zero, and no herd-specific adjustment was applied during prediction. In addition, models developed using all records collected within 70 DIM were also validated using records collected after 70 DIM. This analysis was conducted to assess whether models trained using early-lactation data were transferable to later lactation stages, given that previous work, such as Premi et al. (2021), suggested that several serum biomarkers remain informative beyond early lactation. Notably, only the best model identified from the nested 10-fold random cross-validation procedure was tested for external validation.

Prediction accuracy was evaluated using coefficient of determination (R^2^), RMSE, and ratio of performance to interquartile distance (**RPIQ**). According to Greenberg et al. (2022), a model with RPIQ <1.89 is considered poor, 1.89 to 2.7 is satisfactory, and >2.7 is very good. For BHB and NEFA, predicted values were back-transformed before calculating these metrics. All statistical analyses were performed using R version 4.4.3 (R Core Team, 2026).

Summary statistics for serum biomarkers by stage of lactation are presented in Table 1. The total number of records varied among biomarkers, with BHB, NEFA, and BUN available for nearly all samples (n = 5,904 to 5,909), followed by total protein, albumin, globulin, and AG (n = 5,473), and glucose, calcium, and magnesium (n = 2,149–2,756). Cholesterol, bilirubin, and triglycerides had fewer observations (n = 699–717) from a small number of herds, which should be considered when interpreting the modeling results. As outlined previously, only records collected within 70 DIM were used for model development.

Mean BHB and NEFA concentrations within 70 DIM were 0.67 ± 0.46 and 0.51 ± 0.28 mmol/L, respectively. The NEFA concentration declined after 70 DIM (0.38 ± 0.29 mmol/L), but BHB changed only modestly (0.64 ± 0.24 mmol/L). Similar patterns have been reported, with NEFA concentrations decreasing as cows recover energy balance, but with BHB often showing less pronounced change across lactation (Cattaneo et al., 2023). Blood urea nitrogen concentrations were similar between early and later lactation (5.34 ± 2.08 vs. 5.01 ± 1.94 mmol/L). Although the means of NEFA and BUN within 70 DIM were comparable to those in previous reports (Luke et al., 2019b; Ho et al., 2021), mean BHB was higher in the present study (0.67 vs. 0.54 mmol/L). Consequently, the prevalence of hyperketonemia, defined using a threshold of ≥1.2 mmol/L, was higher in this study than that reported by Ho et al. (2021)—8.5% vs. 2.9%. Similarly, Brunner et al. (2019) showed a relatively high mean BHB concentration (0.7 mmol/L) and a hyperketonemia prevalence of 9.6% in 208 cows from 22 Australian farms, with blood samples collected between 2 and 21 DIM. Further investigation of the data indicated that additional blood samples collected after 2020 had considerably higher BHB concentrations than those collected between 2017 and 2020 in previous studies (0.87 vs. 0.54 mmol/L). This increased BHB appeared to be driven by high prevalence of clinical ketosis in a small number of herds.

Total protein increased from 72.69 ± 6.53 in early lactation to 80.02 ± 6.19 g/L after 70 DIM, driven primarily by globulin (38.35 ± 6.52 to 45.96 ± 7.08 g/L), whereas albumin remained stable (34.34 ± 3.66 vs. 34.06 ± 3.46 g/L). This pattern agrees with earlier observations that globulin concentrations increase postpartum as immune function recovers, whereas albumin is less influenced by lactation stage (Rowlands et al., 1980).

Macromineral concentrations showed modest stage-of-lactation differences. Calcium increased from 2.31 ± 0.22 mmol/L in early lactation to 2.38 ± 0.21 mmol/L after 70 DIM, consistent with recovery of calcium homeostasis following the periparturient period. Magnesium averaged 0.97 ± 0.18 mmol/L in early lactation and 0.91 ± 0.29 mmol/L after 70 DIM. The relatively narrow distributions of Ca and Mg reflect tight homeostatic regulation and may partly explain their limited predictability from MIR spectra.

Among indicators of hepatic function and lipid and carbohydrate metabolism, cholesterol approximately doubled from early to later lactation (2.58 ± 1.14 vs. 4.93 ± 0.99 mmol/L), whereas glucose remained unchanged (3.32 mmol/L in both periods). This strong stage-of-lactation trajectory is consistent with previous studies linking cholesterol to hepatic metabolic status and energy balance (Gross et al., 2015). Bilirubin was slightly lower after 70 DIM, and triglycerides remained low across both stages. Mean haptoglobin concentration in early lactation was 0.20 ± 0.19 g/L, but varied considerably.

Across biomarkers, model performance varied substantially between biomarkers and predictors used (Table 2). It is important to emphasize that MIR spectroscopic predictions are generally indirect, relying on correlations between blood biomarkers and milk components that influence the spectral signal (Eskildsen et al., 2014). In addition, MIR spectroscopy has a limited sensitivity for compounds present at very low concentrations (Dardenne et al., 2015).

**Table 2:** Prediction accuracy of serum biomarkers collected within 70 DIM using MIR spectra, on-farm variables, or both, obtained from nested 10-fold random cross validation^1^

Biomarker^3^	Model^2^											
	1			2			3			4		
	R^2^	RMSE	RPIQ	R^2^	RMSE	RPIQ	R^2^	RMSE	RPIQ	R^2^	RMSE	RPIQ
BHB	0.34	0.38	1.05	0.53	0.32	1.26	0.57	0.30	1.35	0.56	0.31	1.30
NEFA	0.30	0.24	1.49	0.38	0.22	1.59	0.45	0.21	1.69	0.45	0.21	1.69
BUN	0.50	1.47	1.93	0.78	0.99	2.92	0.79	0.92	3.08	0.78	0.98	2.93
Calcium	0.11	0.21	1.12	0.19	0.20	1.17	0.26	0.16	1.38	0.20	0.19	1.17
Magnesium	0.09	0.17	1.06	0.15	0.17	1.09	0.23	0.14	1.33	0.15	0.17	1.09
Protein	0.30	5.48	1.48	0.27	5.58	1.46	0.36	5.01	1.61	0.35	5.28	1.53
Albumin	0.16	3.35	1.27	0.23	3.22	1.33	0.28	2.92	1.45	0.24	3.19	1.33
Globulin	0.25	5.65	1.45	0.22	5.79	1.42	0.29	5.42	1.51	0.29	5.51	1.49
AG	0.18	0.21	1.21	0.18	0.20	1.21	0.26	0.17	1.37	0.22	0.19	1.24
Glucose	0.20	0.60	1.07	0.44	0.50	1.28	0.44	0.38	1.65	0.45	0.50	1.28
Cholesterol	0.77	0.55	1.60	0.63	0.69	1.25	0.76	0.54	1.60	0.76	0.55	1.57
Triglycerides	0.04	0.16	0.80	0.04	0.16	0.80	0.04	0.13	0.92	0.04	0.16	0.79
Bilirubin	0.19	2.28	1.28	0.32	2.10	1.39	0.33	2.05	1.41	0.34	2.07	1.41
Haptoglobin	0.05	0.19	0.56	0.11	0.18	0.58	0.11	0.14	0.70	0.11	0.18	0.58

1 Standard deviations for R^2^, RMSE, and RPIQ were negligible across 50 runs (i.e., maximum of 0.0008) and therefore not presented.

2 Model 1: milk composition + DIM + calving age + breed + herd; model 2: MIR spectra; model 3: MIR + DIM + calving age + breed + herd; model 4: MIR + milk composition + DIM + calving age + breed + herd.

3 NEFA = nonesterified fatty acids; AG = albumin-to-globulin ratio; RMSE = root mean square error; RPIQ = ratio of performance to interquartile distance.

Generally, MIR spectra (model 2) outperformed the model based only on milk composition and on-farm variables, which consisted of DIM, calving age, breed, and herd (model 1). However, the magnitude of improvement differed among biomarkers. Coefficients of determination (R^2^) increased for 9 of 14 biomarkers by 0.06 to 0.28 units, remained unchanged for AG and triglycerides, and decreased slightly for protein, globulin, and cholesterol. Combining MIR spectra with on-farm variables (model 3) produced the highest overall performance, increasing R^2^ over MIR spectroscopy alone for 10 of 14 biomarkers by 0.01 to 0.13 units (3%–62% relative improvement), with no decrease in accuracy for any biomarkers. Similar findings have been reported regarding the primary contribution of MIR spectra as well as additional values of production and management variables to prediction of methane emissions and fertility of dairy cows (Shetty et al., 2017; Shadpour et al., 2022). In contrast, adding conventional milk composition traits (fat, protein, and lactose) to a model already including MIR spectra and on-farm variables (model 4) did not improve accuracy. This outcome was expected because milk composition is predicted from MIR spectra. A similar finding was reported by Ho et al. (2019), who showed that incorporating MIR spectra improved predictive performance for dairy cow fertility, whereas removing milk composition traits did not reduce accuracy. Overall, these results suggest that combining MIR spectra with on-farm variables produced the most effective model for predicting serum biomarkers in dairy cows. Therefore, external validation was conducted using model 3 only.

Regarding individual biomarkers, BHB and NEFA were moderately predicted, with R^2^ values of 0.57 and 0.45, RMSE of 0.30 and 0.21 mmol/L, and RPIQ of 1.35 and 1.69, respectively (Table 3). The most accurately predicted biomarker was BUN (R^2^ = 0.79, RPIQ = 3.08). These results are consistent with previous studies reporting that BHB, NEFA, and BUN are among the most predictable blood biomarkers from milk MIR spectra (Benedet et al., 2019; Bonfatti et al., 2019; Ho et al., 2021). The relatively higher prediction accuracies for BHB, NEFA, and BUN likely reflect their close association with energy and protein metabolism, which influence milk composition and MIR spectral characteristics, whereas minerals, inflammatory biomarkers, and some hepatic biomarkers may have weaker or more indirect relationships with milk spectral information. Luke et al. (2019b), for example, reported R^2^ values of 0.48, 0.61, and 0.90 for serum BHB, NEFA, and BUN, respectively, while these values were 0.63, 0.52, and 0.58 in Benedet et al. (2019). It is important to reiterate that the data used in Luke et al. (2019b) and Ho et al. (2021) were part of that analyzed in this study.

**Table 3:** Prediction accuracy (mean ± SD) of serum biomarkers collected within 70 DIM using MIR spectra, DIM, calving age, breed, and herd (model 3) obtained from external validation^1^

Biomarker^2^	Leave-one-herd-out			Biomarker at >70 DIM		
	R^2^	RMSE	RPIQ	R^2^	RMSE	RPIQ
BHB	0.45 ± 0.17	0.26 ± 0.17	1.13 ± 0.34	0.004	0.33	0.95
NEFA	0.34 ± 0.17	0.28 ± 0.10	1.10 ± 0.36	0.003	0.34	1.29
BUN	0.58 ± 0.22	1.02 ± 0.49	2.01 ± 0.65	0.65	1.36	2.20
Calcium	0.19 ± 0.15	0.18 ± 0.05	1.00 ± 0.22	0.008	0.27	0.80
Magnesium	0.03 ± 0.04	0.15 ± 0.04	0.98 ± 0.16	0.20	0.25	0.99
Protein	0.19 ± 0.09	6.02 ± 0.97	1.15 ± 0.22	0.04	7.91	0.97
Albumin	0.11 ± 0.07	3.67 ± 1.37	1.04 ± 0.29	0.05	3.55	1.32
Globulin	0.16 ± 0.08	5.77 ± 1.05	1.19 ± 0.18	0.09	8.53	1.07
AG	0.11 ± 0.07	0.20 ± 0.07	1.13 ± 0.25	0.07	0.20	0.99
Glucose	0.15 ± 0.11	0.58 ± 0.12	1.01 ± 0.39	0.04	0.75	0.54
Cholesterol	0.13 ± 0.02	0.81 ± 0.37	0.93 ± 0.19	0.09	3.01	0.46
Triglycerides	0.0008 ± 0.0001	0.17 ± 0.03	0.71 ± 0.16	0.07	0.20	0.61
Bilirubin	0.02 ± 0.10	3.11 ± 0.55	1.11 ± 0.41	0.24	3.61	0.42
Haptoglobin	0.10 ± 0.07	0.18 ± 0.05	0.56 ± 0.09	0.08	0.13	0.56

1 Standard deviation of prediction accuracy was not available for validation using records with DIM >70.

2 NEFA = nonesterified fatty acids; AG = albumin-to-globulin ratio; RMSE = root mean square error; RPIQ = ratio of performance to interquartile distance.

Protein fractions (albumin, globulin, and AG), macrominerals (Ca and Mg), and haptoglobin showed only low to moderate prediction accuracy (R^2^ = 0.11–0.36). Glucose and bilirubin were moderately predicted (R^2^ = 0.44 and 0.33, respectively), whereas triglycerides showed negligible predictability (R^2^ = 0.04). The low predictability of triglycerides is consistent with the results of Benedet et al. (2019). Cholesterol achieved relatively high accuracy (R^2^ = 0.76, RPIQ = 1.60); however, a further inspection of the scatter plot between predicted and observed cholesterol revealed 2 distinct clusters corresponding to low and high cholesterol concentrations before and after 30 DIM, respectively, which appeared to largely drive the high overall R^2^. Within each cluster, the R^2^ was approximately 0.25, indicating considerably weaker within-stage predictive ability. This result suggests that the model might have captured broad lactation-stage differences rather than accurately predicting individual-cow variation in cholesterol concentration, despite DIM already being included as a predictor.

Compared with the literature, our glucose, cholesterol, and triglycerides prediction accuracies were similar to those reported by Benedet et al. (2019), with R^2^ = 0.20, 0.16, and 0.39, respectively. In contrast, the prediction accuracies for protein fractions and macrominerals were lower than those reported in some other studies. For example, Soyeurt et al. (2009) reported cross-validation R^2^ values of 0.80, 0.50, 0.70, 0.79, and 0.23 for milk Ca, Mg, Na, P, and K, respectively. However, those results were based on relatively small calibration sets, with only 31 to 57 samples used for mineral reference analyses, which may limit direct comparison with the present study. Giannuzzi et al. (2023) investigated MIR-based prediction of a broader panel of 29 blood biomarkers using machine-learning methods. They reported comparatively good prediction accuracy (R^2^ = 0.34–0.78) for several biomarkers related to energy metabolism, liver function and hepatic damage, inflammation and innate immunity, and some protein fractions, whereas prediction accuracy for oxidative stress indicators and minerals was generally more variable (R^2^ = 0.20–0.78).

Although random cross-validation is commonly used to assess the performance of MIR prediction equations, it often provides overoptimistic results, especially when records from the same herds or same test date are represented in both training and validation sets. Our dataset included 5,936 blood samples from 4,442 cows, which means that some cows contributed more than one record. Wang and Bovenhuis (2019) reported a substantial decline in the prediction accuracy (R^2^) of methane emissions using MIR spectroscopy, from 0.49 to 0.01, on random cross-validation and leave-one-herd-out validation, respectively. This reduction reflects the difficulty of transferring MIR equations across herds differing in management, feeding system, or breed composition. This emphasizes the importance of externally validating the model before practical implementation. In the current study, model transferability was evaluated using both leave-one-herd-out validation and validation in cows sampled after 70 DIM (treated as a single data block), using models trained exclusively on records collected within 70 DIM.

External validation showed clear differences among biomarkers in model robustness across herds and lactation stages. Blood urea nitrogen had the strongest and most consistent external performance, with leave-one-herd-out R^2^ = 0.58 ± 0.22, RMSE = 1.02 ± 0.49 mmol/L, and RPIQ = 2.01 ± 0.65. Performance remained comparable in cows sampled after 70 DIM (R^2^ = 0.65; RPIQ = 2.20). However, the relatively large SD in leave-one-herd-out R^2^ indicates that more data, especially across different feeding and management systems, should be collected to improve the robustness of the model (Pralle and White, 2020). β-Hydroxybutyrate and NEFA showed moderate leave-one-herd-out performance, with average R^2^ values of 0.45 ± 0.17 and 0.34 ± 0.17, respectively. However, their performance was strongly stage-dependent, declining to near zero when applied to cows sampled after 70 DIM (R^2^ = 0.004 and 0.003, respectively). This suggests that these equations captured the early-lactation metabolic signal associated with negative energy balance, lipid mobilization, and ketogenesis, but were not transferable beyond this biological window. Using leave-one-herd-out validation, Gruber et al. (2026) reported MIR BHB prediction accuracies of R^2^ = 0.18 with PLSR and R^2^ = 0.25 with support vector machine or artificial neural network models.

Hepatic, lipid, carbohydrate-related, and inflammatory biomarkers showed poor transferability overall. Glucose had low accuracy in both leave-one-herd-out and >70 DIM validation (R^2^ = 0.15 and 0.04, respectively), and bilirubin, triglycerides, and haptoglobin were not reliably predicted. Haptoglobin showed poor external validity (leave-one-herd-out R^2^ = 0.10 and >70 DIM R^2^ = 0.08). Cholesterol provided the clearest example of overestimation by random cross-validation: despite high random cross-validation accuracy (R^2^ = 0.76), external validation performance was poor (leave-one-herd-out R^2^ = 0.13 and >70 DIM R^2^ = 0.09), suggesting that the apparent accuracy could be driven by DIM-related structure rather than a robust MIR-serum cholesterol relationship. However, it should be noted that the number of herds available for validation differed among traits: bilirubin, cholesterol, and triglycerides were assessed in only 3 herds; glucose in 8 herds; and the remaining biomarkers in 12 to 23 herds. Additional data from more herds would be required for a robust validation.

Overall, external validation confirmed that the MIR-based model for BUN had the most robust and transferable predictive performance, and models for BHB and NEFA showed moderate leave-one-herd-out performance but were limited to early lactation. Models for the remaining biomarkers had low external prediction accuracy. Potential applications of these predictions include monitoring nitrogen use efficiency, ketosis, and energy deficit at the herd and individual-cow level. However, further research is required to evaluate their ability to identify cows exceeding clinically relevant biomarker thresholds and their suitability as practical management tools. In addition, MIR-predicted biomarkers may provide cost-effective phenotypes for future genetic evaluation and genomic selection to improve health and welfare of dairy cows, although further validation of their genetic properties is required before such applications can be implemented.
